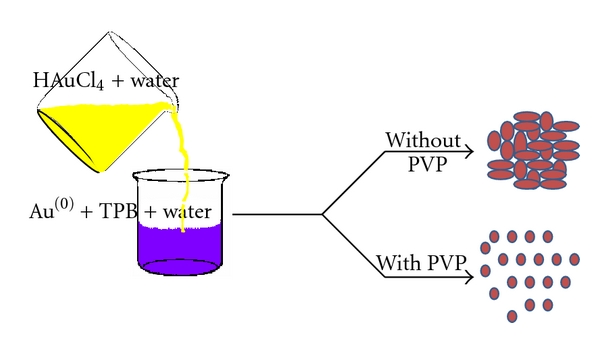# Facile and One Pot Synthesis of Gold Nanoparticles Using Tetraphenylborate and Polyvinylpyrrolidone for Selective Colorimetric Detection of Mercury Ions in Aqueous Medium

**DOI:** 10.1155/2012/348965

**Published:** 2012-03-20

**Authors:** Sidhureddy Boopathi, Shanmugam Senthilkumar, Kanala Lakshminarasimha Phani

**Affiliations:** Electrodics and Electrocatalysis Division, Central Electrochemical Research Institute, Karaikudi 630 006, India

## Abstract

In this work, we reported for the first time, a facile and one step synthesis of gold nanoparticles from HAuCl_4_, employing tetraphenylborate as the reducing agent. The synthesis is not only facile but also yields “dumb-bell-shaped”particles. This shape appears to arise from a possible emulsion of the products of oxidation/decomposition of tetraphenylborate by HAuCl_4_, surrounding the particle. The size and shape of the AuNPs were characterized by Transmission electron microscopy (TEM) and UV-visible Spectroscopy. Interestingly, the addition of polyvinylpyrrolidone (PVP) during the synthesis was found to enhance the stability of the nanoparticle dispersion. The particles synthesized under these conditions assume “spherical” shape with the appearance of only transverse surface plasmon resonance band. The highlight of the observations is that the gold nanoparticles synthesized using tetraphenylborate as reducing agent and PVP as stabilizer are highly stable in alkaline medium, in contrast to the synthesis wherein borohydride is used as reducing agent. The AuNPs synthesized using tetraphenylborate and PVP show their mercury sensing behavior only in the alkaline medium. The color of the nanoparticle dispersion undergoes distinct color change from pink to blue with the addition of mercury ions. They also show dramatic selectivity to mercury ions in presence of other interfering ions, Pb^2+^, Zn^2+^ and Ca^2+^.

## 1. Introduction

 In the recent years, several routes for preparing gold colloids based on the chemical reduction of hydrogen tetrachloroaurate (III) have been discovered [1]. Among them, the classical citrate reduction method has been extensively used for the preparation of aqueous gold nanoparticles with very narrow size distribution. Various other reducing agents have been explored to synthesize AuNPs, which resulted in the discovery of size and shape control during synthesis [2, 3]. Each method confers a special and unique property on the resultant AuNPs, in terms of their physicochemical properties. Because of these unique properties, various groups have paid attention to developing single-step facile methodologies for the synthesis of size- and shape-controlled metal nanoparticles [4–6]. This is because of the reason that the surface plasmon resonance (SPR) of gold nanoparticles varies with size, shape, and the dielectric constant of the surrounding medium [7].

It was earlier discovered that a shift in the SPR of AuNPs acts as a sensing probe for colorimetric detection [8] because the color of the solution changes from red to blue when the analyte is added to the AuNP solution (due to the interacting surface plasmons). There is now an increased research activity in evolving synthetic methodologies to suit the analytical protocols. This type of colorimetric sensors is simple, versatile, and inexpensive and does not need any specialized equipment for the detection of biologically and environmentally important chemical species [9]. Recently, Kim et al. [10] demonstrated AuNPs-based hyper-Rayleigh scattering (HSR) and colorimetric sensing for spectroscopically “silent” heavy metal ions such as lead, cadmium, and mercury; Li and coworkers have shown a method for colorimetric detection of Hg^2+^ ion by DNA-based machine [11]; Mirkin et al. developed a highly selective and sensitive chip-based scanometric assay for the detection of Hg^2+^ using the sharp melting properties of DNA-AuNPs and the selective T-T coordination chemistry for Hg^2+^ [12, 13]. Further, Hirayama et al. have developed a selective colorimetric sensor for Hg^2+^ by using AuNPs-supported thiolated triethylene glycol ligand (Au-S-EG3). They proposed a new mechanism for color change of AuNPs that involves ligand abstraction of gold surface by Hg^2+^ [14]. To summarize, studies on the colorimetric detection of inorganic ions using AuNPs reported thus far essentially belong to two classes, namely, (i) analysis of nonlinear properties of nanoparticles (“plasmonics”) [15] and (ii) chemical derivatization of AuNP surface with ligands selective to the metal ion [12, 13]. 

 In the course of our continuing research on the synthesis of gold nanoparticles through various approaches, we found sodium tetraphenylborate to reduce HAuCl_4_ to Au(0) to form gold nanoparticle dispersions of limited stability. This is understandable as tetraphenylborate is known to be very electron rich and hence expected to reduce metal ions, as observed in the present case. In addition, tetraphenylborate also is prone to oxidation by hexachloroiridate (IV) in a quantitative manner [16]. Tetraphenylborate was earlier found to play a useful role in eliminating the passive oxide formation thus facilitating the direct electrodissolution/redeposition, leading to faceting of metals, for example, gold and silver [17]. To the best of our knowledge, there has been no report on the synthesis of AuNPs using TPB as a reducing agent. Herein we report, for the first time, (a) the synthesis of AuNPs using TPB; (b) stabilization of TPB-derived AuNPs using PVP; (c) a simple label-free colorimetric protocol for selective detection of mercury ions. The work presented here belongs to the class (ii), wherein PVP acts both as a particle stabilizer and selective “ligand” for Hg^2+^ in aqueous medium.

## 2. Experimental Methods and Procedure

### 2.1. Materials

 ZnCl_2_, HgCl_2_, CaCl_2_, Pb(NO_3_)_2_, and HgCl_2_,—E-Merk; tetrachloroaurate (HAuCl_4_), sodium tetraphenylborate (TPB), Nafion solution (5 wt%), Poly(sodium 4-styrenesulfonate) (PSS^−^)—Sigma Aldrich; polyvinylpyrrollidone (PVP)—Sisco Research Laboratories; sodium dodecylsulfonate (SDS)—S. D Fine Chem. Ltd; the aqueous solution was prepared using Milli Q water (18.3 MΩ·cm) grade.

### 2.2. Methods

The UV-vis absorption spectra of the colloidal dispersions were collected on a Cary 500 scan UV-vis-NIR spectrophotometer with incident light normal to the 1 cm path length quartz cuvette. Spectra were collected between the wavelengths of 200 to 1100 nm. TEM examination of the samples was carried out with a Philips CM200 microscope working at 200 kV. A single drop of the aqueous solution of the AuNPs dispersion was placed onto a copper grid coated with a carbon film (400 mesh).The grid was left to dry in air for several hours at room temperature. Cyclic-voltammetric experiments were carried out using a Potentiostat BAS 100 B (Bioanalytical Systems Inc.) at ambient temperature (25 ± 1°C). For voltammetric studies, a glassy carbon working electrode of area 0.07 cm^2^ (BAS Inc.), a platinum foil auxiliary electrode, and an mercury-murcurous sulphate (MSE) reference electrode were used.

### 2.3. Procedure

 Sodium tetraphenylborate (TPB) was dissolved in water under sonication to prepare a 1 mM solution. To a 10 mL solution of 1 mM TPB, aliquots of 25 mM HAuCl_4_ solution, namely, 100, 200, 300, 400, and 800 *μ*L, were added in separate beakers. The concentration of HAuCl_4_ in these solutions works out to be 0.25, 0.5, 0.75, 1.0, and 2.0 mM, respectively. During the addition of HAuCl_4_ to 1 mM TPB, the colour of the solution changes immediately from colourless to blue. These mixtures were kept for standing for about one hour, after which different colours of AuNPs were formed in the containers.

In the case of the preparation of the stabilized AuNPs using TPB as the reducing agent, the same experimental conditions followed above were employed using stabilizers such as polyvinylpyrrolidone (PVP), sodium polystyrene sulfonate (PSS), Nafion 117, and sodium dodecylsulfate (SDS). In a typical preparation, the stabilizer (0.01% w/v ratio) was mixed with 1 mM TPB solution and then 1 mM HAuCl_4_ solution was added. In presence of the stabilizers, the colour of the solution is pink after the reaction is complete.

For the experiments on colorimetric sensing, PVP-stabilized AuNP solutions were used. A volume of 700 *μ*L of the PVP-stabilized AuNP solution, diluted to 10 mL in water, was added to 600 *μ*L of 0.1 M NaOH to adjust pH to 12. When an aliquot of 500 *μ*L solutions of different metal ions, such as ZnCl_2_, CaCl_2_, Pb(NO_3_)_2_, and HgCl_2_, was added in different beakers containing the PVP-stabilized AuNPs, only Hg^2+^ was found to change the pink colour of the solution to blue whereas the other metal ions do not change the colour. 

## 3. Results and Discussion

**Figure 1:**
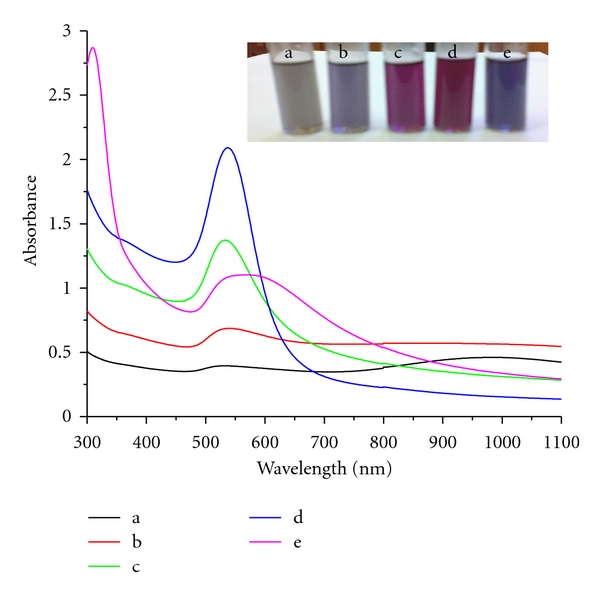
UV-vis spectra of AuNPs of various concentration (a) 0.25 × 10^−3^, (b) 0.50 × 10^−3^, (c) 0.75 × 10^−3^, (d) 1.00 × 10^−3^, and (e) 2.00 × 10^−3^ M of HAuCl_4_ solution using 1 × 10^−3^ M TPB as reductant. *Inset*. Photographic images show different colors of AuNPs in various concentrations such as (a) 0.25 × 10^−3^ M, (b) 0.50 × 10^−3^ M, (c) 0.75 × 10^−3^ M, (d) 1.00 × 10^−3^ M, and (e) 2.00 × 10^−3^ M of HAuCl_4_ solution using 1 × 10^−3^ M TPB as reductant.

 When a solution of HAuCl_4_ was added to sodium tetraphenylborate (1 × 10^−3^ M), the initially colorless solution changed to blue immediately. On standing for one hour, the blue colour is changed to various colours of AuNPs, depending on the concentration of HAuCl_4_ (AuNPs in the sample bottles marked a, b, c, d, and e in the increasing order of HAuCl_4_ concentration) (inset, Figure 1). The formation of AuNPs was indicated by the appearance of an absorption maximum at wavelengths of 527, 540, 533, 537, and 569 nm for increasing concentration of HAuCl_4_, 0.25, 0.50, 0.75, 1.0, and 2.0 mM, respectively, that are shown in Figure 1. It is observed that the intensity of the transverse surface plasmon resonance, absorption of AuNPs increases with increasing concentration of HAuCl_4_ at a fixed concentration of TPB, except in the case where HAuCl_4_ is in excess of TPB concentration. The additional absorption peak at 982 nm, due to the longitudinal component of plasmon resonance, was observed, for the HAuCl_4_ concentrations of 0.25 and 0.50 mM. For the other concentrations of HAuCl_4_, namely, 0.75 and 1.0 mM, the longitudinal component is completely absent. For HAuCl_4_ concentrations in excess of TPB, only a broad peak at 569 nm is seen. The absorption in two different wavelength regions can now be explained taking the size and shape of the gold nanoparticles into consideration. In general, the size effects are reflected in the absorption spectrum, with surface plasmon resonance slightly shifting to higher wavelength values as the particle size increases, due to the aggregation of nanoparticles [18]. In addition, the formation of nonspherical particles with a higher aspect ratio can result in the red shift of the absorption maximum. Hence, the above results point to the possibility of AuNPs of the samples in a, b, and e being of bigger size and in an elongated form, but the AuNPs in c & d are relatively smaller in size for which the longitudinal component is absent. The formation of AuNPs, from equal concentrations (1 mM) of HAuCl_4_ and TPB, was followed by UV-vis spectroscopy at various time intervals (see Figure S-1 in Supplementary Material available online at doi: 10.1155/20/2/348965). After the addition of HAuCl_4_, absorption at 537 nm increases gradually with time and after a period of one hour, the peak does not grow in intensity. This indicates that the reduction of Au^3+^ to AuNPs occurs instantaneously and is associated with a fast rate of formation.

**Figure 2:**
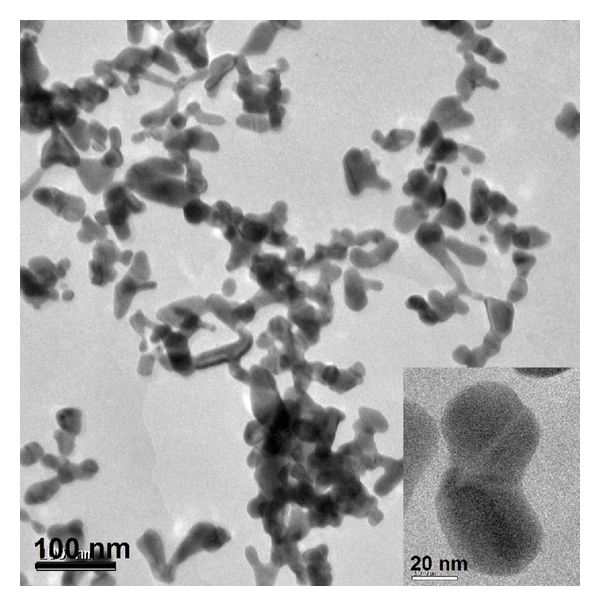
Transmission electron microscopy of AuNPs synthesized by TPB act as reductant *Inset*. Higher magnification of AuNPs.

 In order to verify the possibility of formation of particles in an elongated form, TEM images of the samples synthesized using equimolar concentrations of auric acid and TPB (each 1 mM) were collected (Figure 2), in which dumbbell shaped particles of average diameter of ~15 nm can clearly be seen. In a clear description, this shape can be explained as due to the joining of two spherical particles by a “sheath” of some organic matter. The origin of this “sheath” may be from the byproducts formed during the oxidation of TPB by HAuCl_4_. When a strong oxidant like auric acid is added to TPB solution, the pH of the solution drops below 3.0 and hence TPB can undergo decomposition to form benzene, biphenyl and borate anions [19]. During this process, AuCl_4_^−^ is reduced to Au(0). Incidentally, oxidation of TPB by hexachloroiridate (IV) was earlier reported by Abley and Halpern [16] in which TPB undergoes oxidation to form biphenyl mainly and other species. During this process, Ir (IV) is reduced to Ir (III). Taking into account the above presented results and the available information from literature [16, 20], the following representative mechanism can be assigned to the reduction of Au^3+^ to Au^0^ by TPB:

(1) $$\begin{array}{cc} & \text{TPB}+{\text{H}}_{2}\text{O}+{\text{H}}^{+}+{{\text{AuCl}}_{4}}^{-}\\ & \;\;\to \text{phenylboronic}\;\text{acid}+\text{benzene}+\text{biphenyl}+\text{Au}\left(0\right). \end{array}$$

**Figure 3:**
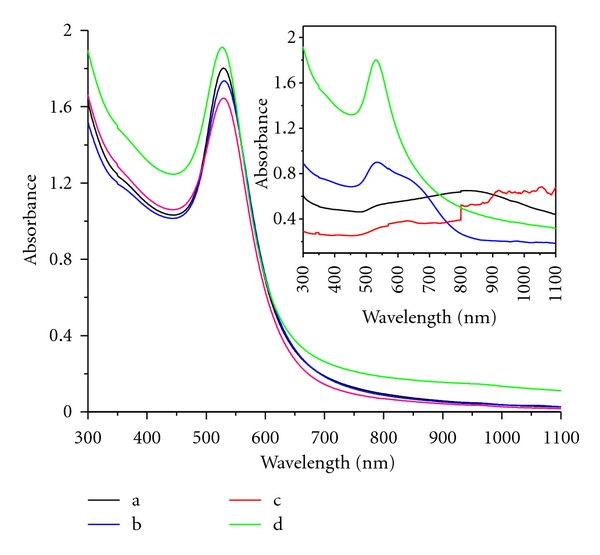
UV-vis spectra of AuNPs synthesized using various stabilizers: black line: PSS^−^, blue line: Nafion, red line: SDS^−^ and light green line: PVP; *Inset*. UV-vis spectra of various stabilized AuNPs such as black line: PSS^−^, blue line: Nafion, red line: SDS^−^, and light green line: PVP by addition of 3 M NaCl.

Further, the byproducts of this reaction, namely, benzene/biphenyls, may form an “emulsion” with interfacial water [21]. It is believed that this “emulsification” is responsible for the controlled growth and stabilization of AuNPs. Though the emulsion formation is visually observed, further characterization is not attempted in the present work. Interestingly, the appearance of this shiny blue-colored emulsion bears similarity to liquid crystalline dispersions. As benzene/biphenyl-water emulsion is associated with poor stability, the particles aggregate and sediment at the bottom of the container over a period of time. During our attempts to improve the particle stability using stabilizers, we found polyvinylpyrrolidone (PVP) to not only stabilize the particle, but to also confer chemical sensing ability to the AuNPs. Other stabilizers studied include 0.01 (w/v%) of sodium dodecylsulfate (SDS), sodium polystyrenesulfonate (Na-PSS), and Nafion 117 and they were added to solutions containing auric acid and tetraphenylborate. All the stabilized AuNPs exhibit sharp transverse surface plasmon resonance absorption at 529 nm alone without any contribution from longitudinal surface plasmon, indicating the formation of Au nanoparticles without aggregation (Figure 3). These dispersions containing PVP, Nafion, and Na-PSS are found to be resistant to the usual destabilizing action of NaCl electrolyte at 3.0 M concentration (inset, Figure 3). However, SDS-containing dispersion appears to be destabilized as seen visually and in the UV-vis spectrum (inset, Figure 3). 

**Figure 4:**
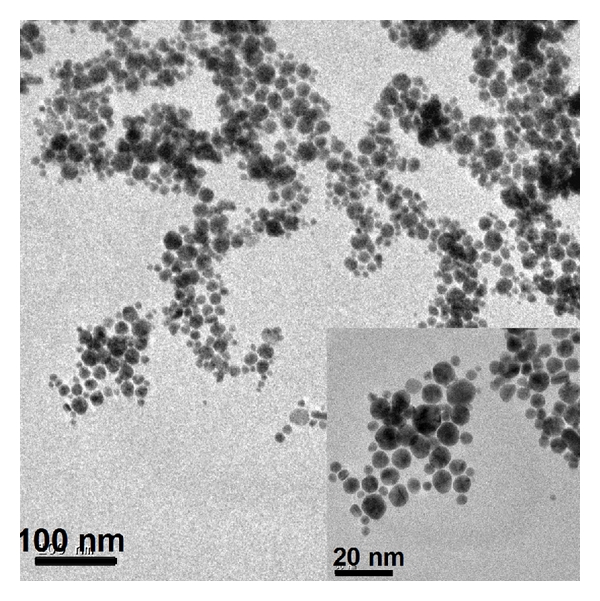
Transmission electron microscopy of PVP stabilized AuNPs synthesized by TPB act as reductant; *Inset.* Higher magnification of PVP stabilized AuNPs.

 It is noticed that PVP-stabilized AuNPs only show absorption at 529 nm without any shift of SPR. The Nafion and PSS-stabilized particles exhibit both transverse- and longitudinal components of surface plasmon resonance. These experiments show that PVP is capable of stabilizing gold nanoparticles, due to the lone pair of electrons from nitrogen and oxygen atoms in the polar groups of PVP repeating unit, interacting with AuNPs [22]. For all concentrations of auric acid in solutions containing a fixed concentration of PVP and TPB, only the transverse surface plasmon resonance absorption peak at 529 nm appears (see figure S-2 in supporting information). This behaviour is in contrast to that of the case in which PVP is absent. This clearly indicates that there is no aggregation of AuNPs when PVP is used. A typical TEM image obtained with PVP shows that all the AuNPs are spherical in shape, polydisperse with respect to their size with an average diameter of the particles around 5 nm (Figure 4). The size range observed in TEM is in correspondence with the location of plasmon resonance absorption in the UV-vis spectroscopy, as discussed above. 

To elucidate the mechanism of formation of gold nanoparticles by the addition of HAuCl_4_ to TPB solutions, cyclic voltammetric studies were carried out. The cyclic voltammetric response of TPB at a glassy carbon electrode in 0.5 M H_2_SO_4_ solutions containing PVP, with and without dissolved HAuCl_4_ is shown in Figure S-3 in the supporting information. Three irreversible peaks at 0.024, 0.62, and 0.98 V versus MSE are observed without the added HAuCl_4_ [20]. Upon the addition of HAuCl_4_ to this solution, all these peaks disappear, thus indicating the consumption of the electroactive TPB through decomposition. All the observations presented for the formation of AuNPs through this method could be explained using Scheme 1. 

In an exploration to find applications for these PVP-stabilized AuNPs, we turned our attention to colorimetric detection of metal ions in aqueous media, as the color of AuNPs prepared through the above route is stable and can be sensitive to changes in environment. This enables one to examine the chemical sensing behavior in response to the metal ion content. A number of colorimetric-based sensors for the detection of Hg^2+^ ion have been reported in recent times [10–15]. With this in mind, we have shown the colorimetric sensing of Hg^2+^ ion sensor using AuNP_TPB-PVP_. An important observation that deserves mentioning here is the stability of the AuNP_TPB-PVP_ in solutions of high electrolyte concentration and alkaline pH. In typical experiments, aliquots of 10 *μ*M of HgCl_2_ solution were then added to the AuNP_TPB-PVP_ (pH = 12) (pH was adjusted by adding NaOH solution), to observe changes in the UV-vis absorption behaviour. With the addition of HgCl_2_ solution, the initial pink color instantaneously changes to blue; the transverse surface plasmon resonance absorption peak at 529 nm slightly shifts to 540 nm with increasing absorbance. The shift of the absorption peak can arise from the aggregation of gold nanoparticles. Change of shape of the particles does not appear to happen as absorption due to longitudinal surface plasmon resonance is absent. With each addition of Hg^2+^, the intensity of blue color increases. The development of blue color was found to depend on factors such as the concentration of PVP and AuNP solution pH.

**Figure 5:**
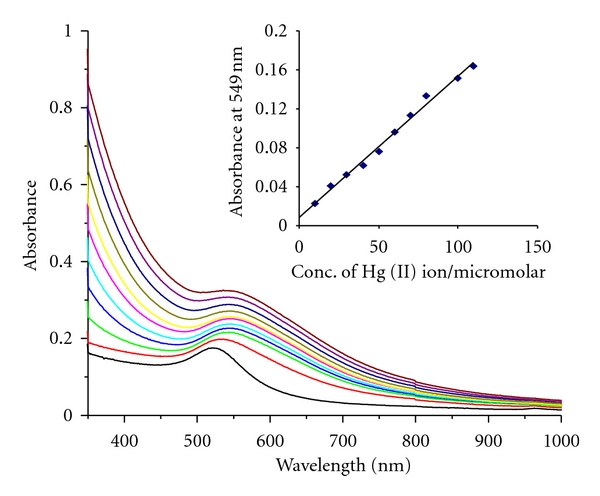
UV-vis spectra of PVP stabilized AuNPs (pH = 12) before and after each addition corresponding to 10 *μ*M Hg^2+^ ion; *Inset.* Calibration plot of intensity of absorption value versus Hg^2+^ ion concentrations for each addition of 10 *μ*M.

**Figure 6:**
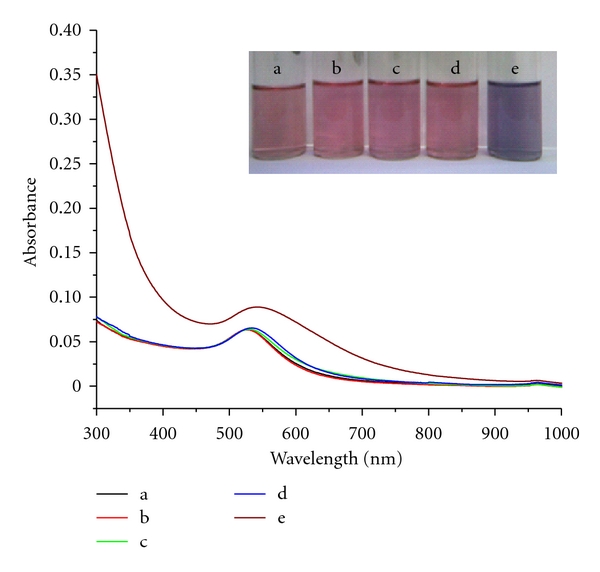
UV-vis spectra of PVP stabilized AuNPs (pH = 12) with various metal ions; (a) no metal ion (blank), (b) Pb^2+^ (c) Ca^2+^ (d) Zn^2+^ and (e) Hg^2+^; *Inset.* Photographic images show various metal ion in PVP-stabilized AuNPs (pH = 12); (a) no metal (blank), (b) Pb^2+^, (c) Ca^2+^, (d) Zn^2+^, and (e) Hg^2+^ ions.

 In order to obtain instant changes on the addition of Hg^2+^, the conditions with respect to PVP concentration and solution pH were optimized that worked out to be PVP (0.001% w/v ratio) and pH = 12. It is important to note that under conditions of pH < 10, the color change to blue is time consuming, sometimes the time duration exceeding an hour for the color change to be observed. Moreover, PVP concentration is also an important criterion for the colorimetric detection of mercury ion. When we used a high concentration of PVP, the change from pink to blue color is more time consuming though solution at pH = 12. Hence was optimized the concentration of PVP (0.001% wt/v ratio) for the colorimetric detection of mercury ion. Invariance of the plasmon resonance peak at 527 nm with changes in PVP concentration and alkaline pH was useful in ensuring the sensitivity of the solution to metal ion alone. In contrast to the PVP-stabilized AuNPs synthesized using TPB as reducing agent, the ones obtained by borohydride reduction were found to be highly unstable under alkaline pH, precluding the possibility of using them for metal ion sensing. This shows the distinct advantage with the AuNPs synthesized using TPB and PVP. Interestingly, the intensity of blue color increases with the concentration of the added mercury ion. Hence, it is believed that high alkaline conditions favour displacement of PVP from the surface of AuNPs. Using AuNP_TPB-PVP_ at pH = 12, the color changes that occur on the addition of metal ions are followed spectrophotometrically (Figure 5). The plot of absorbance versus concentration of Hg^2+^ ion shows a perfect linear relationship with a correlation coefficient value of 0.992 and the detection limit of 10 *μ*M (inset, Figure 5). Having established the fact that Hg^2+^ can be sensed using AuNP_TPB-PVP_, it is appropriate to examine the effect of other interfering metal ions (e.g., Pb^2+^, Zn^2+^ and Ca^2+^ that are present in water) on the sensing behaviour of these nanoparticles. As can be seen from Figure 6, the addition of these metal ions does not affect the surface plasmon absorption of the AuNPs. It can also be visually noticed (digital photographs in inset, Figure 6) that the pink color of the solution is unaffected on the addition of Pb^2+^, Zn^2+^, and Ca^2+^. Hence, two features of this study become clear: (a) surface plasmon absorption of AuNP_TPB-PVP_ can be used for monitoring the concentration of Hg^2+^; (b) an excellent selectivity of AuNP_TPB-PVP_ for Hg^2+^ ions in presence of other metal ions. This selectivity can be rationalized by taking cues from the studies on the preference of PVP for Hg^2+^, to other metal ions [23]. An observation that distinguishes the present method from others is that the colorimetric sensing of mercury is not associated with aggregation-related changes in the longitudinal surface plasmon band position. Instead, only the position of the transverse SPR band is monitored for ion sensing, as the initial particles are spherical in shape. The nonemergence of longitudinal band with the addition of mercury ions confirms the retention of spherical shape. The scheme developed in this work is elegant and simple to the best of our knowledge. An important consideration in the development of chemical sensing mechanisms is how low the detection limit one could reach. The detection limit found in these preliminary investigations is approximately 10 *μ*M. In order to improve the sensitivity to nanomolar levels, one needs to increase the depth of color changes that warrant further studies.

 In summary, the following novel features are shown in this work: (a) facile synthesis of stable gold nanoparticles using a hitherto unknown reductant, tetraphenylborate (despite its low solubility in aqueous solutions); (b) tetraphenylborate, while reducing HAuCl_4_ to Au(0) is decomposed to form dispersions *akin* to liquid crystalline media; (c) the stability of gold nanoparticles formed on reduction by TPB can further be enhanced by protection using PVP; (d) PVP effects change of the shape of the particles from initially dumbbell to polydisperse spherical shape of much lower particle size (approx. 5 nm) (Figure 4), as indicated by the disappearance of the longitudinal surface plasmon absorption in the spectrum; (e) as the AuNP surface is covered by a thin layer of PVP (likely a few monolayers), the latter is susceptible to its removal by metal ions, specifically Hg^2+^; (f) surface plasmon absorption of AuNP_TPB-PVP_ can be used as an indicator of the presence of Hg^2+^ which can be followed by UV-vis spectroscopy. What now remain to be understood are the mechanism of resistance of AuNP_TPB-PVP_ to alkaline pH and selectivity to Hg^2+^. Current investigations are focused on these issues.

## Supplementary Material

Figure S1: 3 UV-vis spectra obtained during the formation of AuNPs at different time intervals after addition of HAuCl4 to the solution containing TPB *(*both concentrations is 1.00 × 10^*‑*3^ M).
Figure S2: UV-vis spectra of PVP stabilized AuNPs of various concentration *(*a) 0.25 × 10^*‑*3^, *(*b) 0.50 × 10^*‑*3^, *(*c) 0.75 × 10^*‑*3^, *(*d) 1.00 × 10^*‑*3^, and *(*e) 2.00 × 10^*‑*3^ M of HAuCl_4_ solution using 1 × 10^*‑*3^ M TPB as reductant.
Figure S3: Cyclic voltammetry of TPB *(*1.00 x10^*‑*3^), PVP *(*0.01g) dissolved in 0.5M H_2_SO_4_ solution containing with *(*blue color line) and without *(*black color line) HAuCl_4_.

## Figures and Tables

**Scheme 1:**