# Supplementary material for: Facile and One Pot Synthesis of Gold Nanoparticles Using Tetraphenylborate and Polyvinylpyrrolidone for Selective Colorimetric Detection of Mercury Ions in Aqueous Medium
Source: J Anal Methods Chem. 2012 Mar 20;2012:348965. doi: 10.1155/2012/348965 (PMC3335305; doi:10.1155/2012/348965)
Supplement: Supplementary file 1 — Figure S1: 3 UV-vis spectra obtained during the formation of AuNPs at different time intervals after addition of HAuCl4 to the solution containing TPB (both concentrations is 1.00 × 10‑3 M). Figure S2: UV-vis spectra of PVP stabilized AuNPs of various concentration (a) 0.25 × 10‑3, (b) 0.50 × 10‑3, (c) 0.75 × 10‑3, (d) 1.00 × 10‑3, and (e) 2.00 × 10‑3 M of HAuCl4 solution using 1 × 10‑3 M TPB as reductant. Figure S3: Cyclic voltammetry of TPB (1.00 x10‑3), PVP (0.01g) dissolved in 0.5M H2SO4 solution containing with (blue color line) and without (black color line) HAuCl4. [file 348965.f1.pdf]

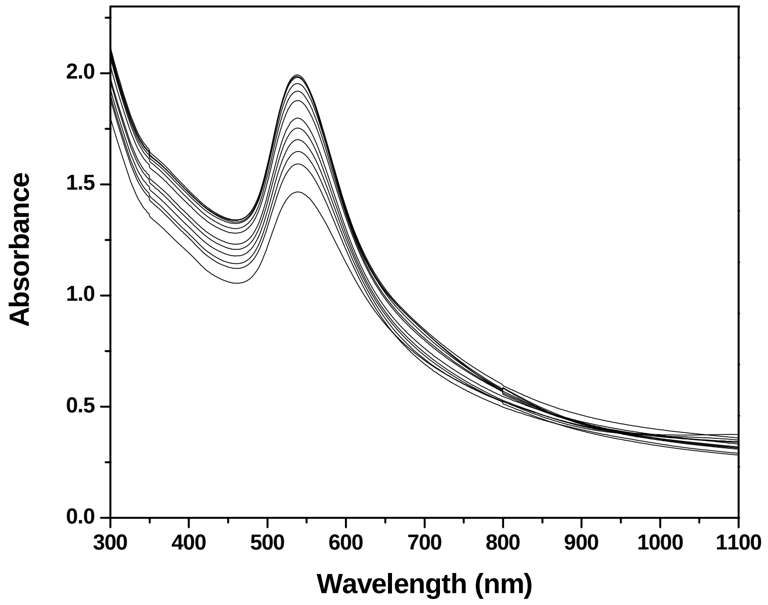

S1.-3 UV-vis spectra obtained during the formation of AuNPs at different time intervals after addition of HAuCl4 to the solution containing TPB (both concentrations is 1.00×10^{-3} M)

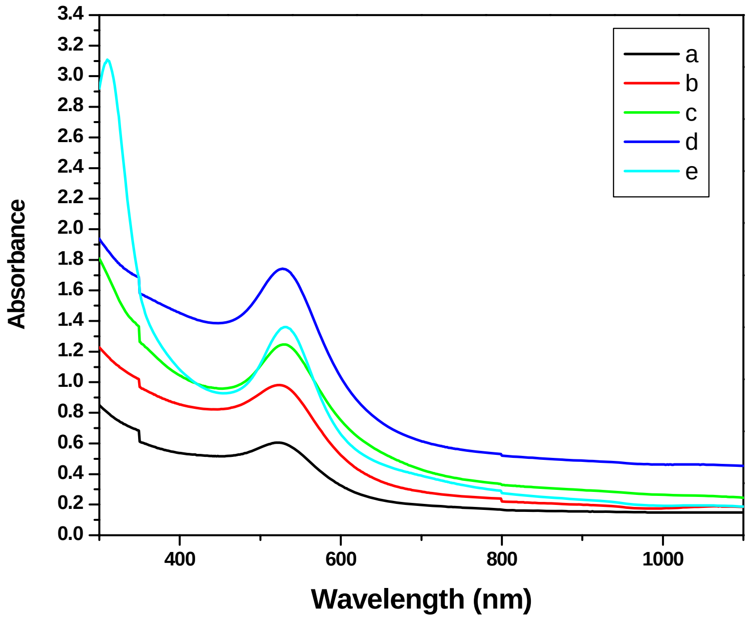

S-2. UV-vis spectra of PVP stabilized AuNPs of various concentration (a) 0.25 x 10^{-3}, (b) 0.50 x 10^{-3}, (c) 0.75 x 10^{-3}, (d) 1.00 x 10^{-3}, and (e) 2.00 x 10^{-3} M of $HAuCl_4$ solution using 1 x 10-3M TPB as reductant.

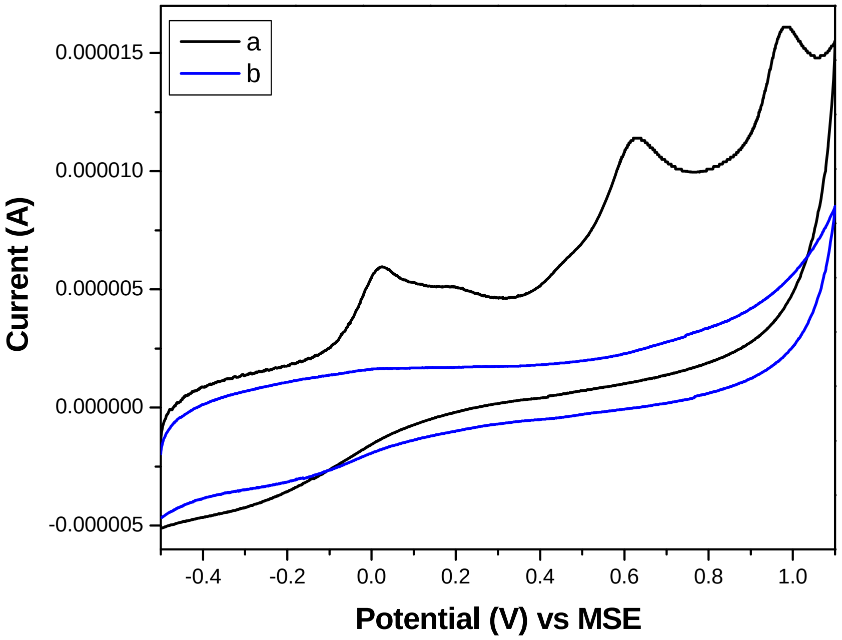

S-3 Cyclic voltammetry of TPB (1.00 x10^{-3}), PVP (0.01g) dissolved in 0.5M H_2SO_4 solution containing with (blue color line) and without (black color line) $HAuCl_4$
